# Investigation into the Internal Factors for the Catalytic Oxidation of Cyclohexane by Zr(IV)-Based Metal-Organic Frameworks

**DOI:** 10.3390/polym16223114

**Published:** 2024-11-06

**Authors:** Kechao Wang, Zhi-Peng Tao, Jia-Qi Chu, Shi-Ming Wang, Zheng-Bo Han

**Affiliations:** 1 College of Chemistry, Liaoning University, Shenyang 110036, China; kchaowang@163.com (K.W.); taozhipeng1996@163.com (Z.-P.T.); 17862705718@163.com (J.-Q.C.); 2 College of Light Industry, Liaoning University, Shenyang 110036, China

**Keywords:** MOFs, active sites, cyclohexane, substrate enrichment

## Abstract

Three porous Zr(IV)-based MOFs, UiO-66, MOF-808 and MOF-802, were selected to catalyze cyclohexane oxidation in order to reveal the intrinsic factors of the active site and catalytic performance. It was found that reducing the number of Zr_6_O_8_ ligand linkages could improve the catalytic efficiency of cyclohexane oxidation. The main reason for this is the different enrichment abilities of MOFs with different linkage numbers for cyclohexane, which was confirmed by the TPD of cyclohexane and also by GCMC simulations. Meanwhile, the catalytic effect of MOF-802 was lower than expected due to its low porosity and narrow inner pore size. The by-products were identified in detail by GC-MS, providing evidence for this catalytic mechanism. In addition, the potential of this catalyst for industrial applications in cyclohexane oxidation was demonstrated by optimizing the catalytic conditions.

## 1. Introduction

Metal-organic frameworks (MOFs) are currently very popular crystalline materials constructed from metal ions/clusters and organic ligands, and form 1-, 2-, or 3-dimensional structures [1]. MOFs have been shown to have a multitude of applications due to their exceptional performance in catalysis [2,3,4], sensing [5,6,7], gas storage and separation [8,9], and adsorptive removal of hazardous materials [10]. This is attributed to their distinctive physicochemical properties, which include adjustable inner pore sizes, extensive internal surfaces, remarkably high porosity, and a plethora of active functional sites. Especially in the field of catalysis, MOFs are typically endowed with higher catalytic activity either through covalent incorporation of catalytically active species into the organic ligands or the inorganic nodes or through entrapment of metal nanoparticles, enzymes, or inorganic clusters in the channels. Nevertheless, some of the transition metals are very active and have been reported to have applications in many catalytic fields, such as MIL-100 [11], MIL-101 [12], MOF-74 (Mn) [13], and so on. In addition to the intrinsic properties of the metal, the active sites of MOFs play a pivotal role in the catalyst process. By exposing a greater number of active sites, the catalytic performance of MOFs can be markedly enhanced. Nevertheless, there is a dearth of comprehensive studies elucidating the underlying mechanisms responsible for this performance improvement.

Zirconium-based MOFs (Zr-MOFs) are among the foremost classes of MOFs for applications in chemical catalysis. By far the most ubiquitous subset of Zr-MOFs is that based on hexa-zirconium (IV)-oxo, hydroxo, aqua nodes, and carboxylate-terminated linkers. Zr_6_O_8_ nodes can accommodate 12 linker-connected carboxylates, such as UiO-66 [14], 10 linker-connected carboxylates, such as DUT-69 [15] and MOF-802 [16], 8 linker-connected carboxylates, such as NU-1000 [17] and MOF-545 [18], and 6 linker-connected carboxylates, such as MOF-808 [16] and PCN-224 and so on [19]. When they are used in catalytic processes with Zr_6_O_8_ as the catalytic activity center, they often show large differences in catalytic activity. Lin et al. tuned the Lewis acidity of MOFs via perfluorination of bridging ligands to reveal the influence of acidity of Zr_6_O_8_ on catalytic performance [20]. Wang et al. reported that hybrid UiO-66-X (X = NO_2_, NH_2_, H) MOFs can exhibit different electronic properties of Zr-oxo nodes while retaining similar crystal structures after modification by various ligands. The influence of electronic density of Zr_6_O_8_ on catalytic performance was revealed [21]. Nevertheless, the underlying cause of the discrepancy in catalytic performance resulting from the variation in the number of connection linkers in Zr_6_O_8_ remains unclear.

Bearing these in mind, three representative Zr-MOF catalysts, 6-linker-connected MOF-808, 10-linker-connected MOF-802 and 12-linker-connected UiO-66, were selected in our study to catalyze cyclohexane oxidation using different connected Zr_6_O_8_ clusters as metal nodes to reveal the internal reasons for the differences in catalytic performance. The experimental results showed that the catalytic activity decreased significantly with the increase in the number of Zr_6_O_8_ connections. By means of TBD characterization and GCMC simulation, we confirmed that the reason for this discrepancy lies in the different ability of catalysts with different numbers of Zr_6_O_8_ connections to enrich substrates. Accordingly, the catalytic conditions were optimized, and the optimal catalysis performance and the reaction mechanism of MOF-808 were further investigated. The experimental results demonstrated that the catalytic performance of the catalyst surpassed the current industrial catalytic level (~5% conversion), indicating its potential value in industrial applications [22].

## 2. Experimental Section

### 2.1. Chemicals

Zirconium chloride octahydrate (ZrOCl_2_·8H_2_O, AR (Analytical Reagent), Bidepharm, Shanghai, China), zirconium chloride (ZrCl_4_, AR, Macklin, Shanghai, China), 3,5-pyrazoledicarboxylic acid monohydrate (PZDC, AR, Macklin, Shanghai, China), benzene-1,3,5-tricarboxylic acid (H_3_BTC, AR, Macklin, Shanghai, China), tert-Butyl hydroperoxide solution (TBHP, AR, Macklin, Shanghai, China), and phthalic acid (H_2_BDC, AR, Macklin, Shanghai, China) were used. Ethanol (C_2_H_5_OH, AR, Sinopharm Chemical Reagent Co., Ltd., Shanghai, China), formic acid (HCOOH, 99.99%, Tianjin Damao Chemical Co., Ltd., Tianjin, China), N,N-Dimethylpropanamide (DMF, AR), acetonitrile (CH_3_CN, AR) and acetic acid (CH_3_COOH, AR) were supplied by Tianjin Fuyu Chemical Co., Ltd. (Tianjin, China). All reagents were purchased and used without further purification.

### 2.2. Catalyst Syntheses

Preparation of MOF-808: MOF-808 was synthesized on the basis of recently published articles with slight modifications [23]. Briefly, H_3_BTC (1191 mg, 6.0 mmol), acetic acid (60 mL), H_2_O (60 mL), and concentrated HCl (600 µL) were added to a round-bottom flask whilst stirring. The above-mixed liquid was named as the ligand precursor solution. Then, ZrOCl_2_⋅8H_2_O (5616 mg, 18.0 mmol) was added to it. The mixture was heated in an oil bath and stirred at 110 °C under reflux for 24 h. Finally, the product of MOF-808 was dried in a vacuum oven at 358 K overnight.

Preparation of MOF-802: MOF-802 was synthesized on the basis of recently published articles with slight modification [24]. A 50 mL vial was loaded with PZDC (348 mg, 2 mmol) and ZrOCl_2_·8H_2_O (560 mg, 1.74 mmol), and completely dissolved in a solvent mixture of DMF/formic acid (6 mL/4 mL); the mixture was heated at 130 °C for 3 days. Finally, the product of MOF-802 was dried in a vacuum oven at 358 K overnight.

Preparation of UiO-66: UiO-66 was synthesized on the basis of recently published articles with slight modifications [25]. A 50 mL vial was loaded with BDC (123 mg, 0.75 mmol) and ZrCl_4_ (126 mg, 0.54 mmol), and completely dissolved in a solvent mixture of DMF/concentrated HCl (30 mL/2 mL); the mixture was heated at 120 °C for 24 h. Finally, the product of UiO-66 was dried in a vacuum oven at 358 K overnight.

### 2.3. Characterizations

Powder X-ray diffraction (PXRD) analysis was performed on a Bruker AXS D8 advance X-ray diffractometer using Cu Kα radiation (λ = 0.15406 nm) at an accelerating voltage of 30 kV (Bruker, Ettlingen, Germany). The X-ray photoelectron spectrometer (ThermoFischer, ESCALAB 250Xi, Waltham, MA, USA) was used for this experiment, and the XPS data were calibrated using a standard C 1s binding energy of 284.8 eV. The N_2_ adsorption–desorption isotherms were measured at 77 K using a specific surface area analyzer (BeiShiDe 3H-2000PS1, Beishide Instrument Technology Co., Ltd., Beijing, China). Qualitative analysis was performed using GC-MS (Tsushima, Japan). Quantitative analysis was performed using GC (Agilent Technologies 6890N, Agilent Technologies, Inc., Santa Clara, CA, USA). IF-IR and Roman data were collected using a Nicolet 5DX instrument (Nicolet Instrument Corporation, Madison, WI, USA) and a commercial Xplora plus Raman spectrometer (HORIBA Instruments, Co., Ltd., Shanghai, China) at room temperature, respectively. Cyclohexane temperature programmed desorption (TPD) was tested using an Automatic Chemisorption Analyzer (Bayside instrument, BSD-C200, Beijing, China) with an activation temperature of 100 °C and a purge with He, with adsorption and purge durations of 30 min and 60 min, respectively. The ramp rate was 10 °C/min.

### 2.4. Catalytic Test

Cyclohexane 1 mL, CH_3_CN 4 mL, and TBHP 1 mL were added to a 25 mL Schlenk tube. The system air was exhausted with an oxygen bulb so that the reaction system was always in a high-purity O_2_ atmosphere. The reaction was heated with stirring in an oil bath at different temperatures to the target time. At the end of the reaction, the reaction solution was removed and the catalyst was centrifuged out. Then, 20 μL of the upper solution was taken and diluted with 40 μL of anhydrous methanol.

### 2.5. Grand Canonical Monte Carlo (GCMC) Simulations

GCMC simulations were carried out to explore the distribution of the possible adsorption sites of the cyclohexane molecule within the MOF frameworks. The forcite module was used to optimize the unit cell and the guest molecules. A 1 × 1 × 1 supercell was used as the simulation box.

### 2.6. Free-Radical Capture Methods

For free-radical trapping experiments, an additional 20 μmol of free-radical shielding agent was added, and the rest of the conditions were kept the same.

## 3. Results and Discussion

### 3.1. Characterization of Catalysts

**Scheme 1:**
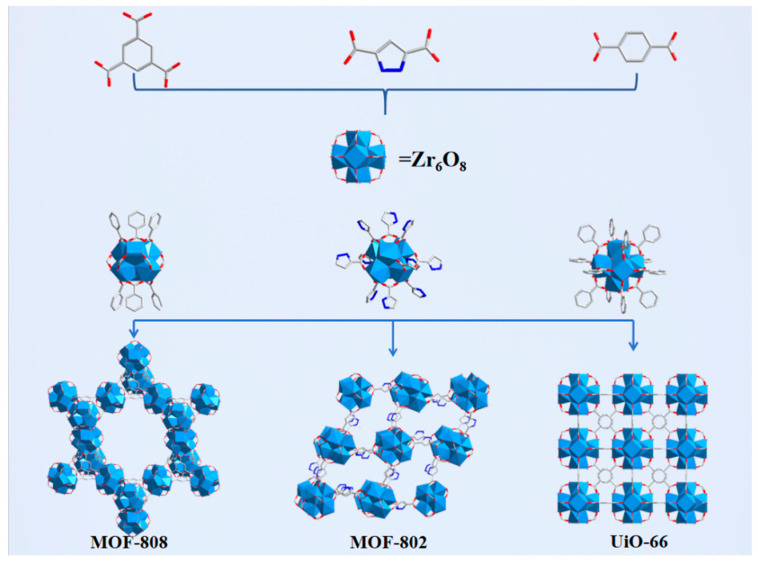
Schematic diagram of the structure of three Zr-MOFs (red: O, blue: N, gray: C).

**Figure 1:**
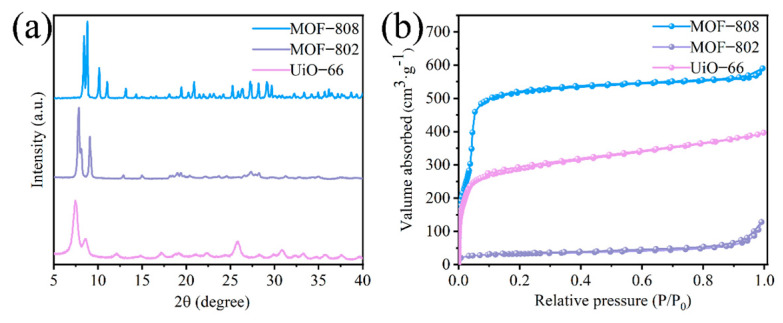
(**a**) PXRD patterns, and (**b**) Nitrogen sorption isotherms at 77 K for MOF-808, MOF-802 and UiO-66.

Three Zr_6_-node-classical MOFs, including MOF-808, MOF-802 and UiO-66, were constructed from hexanuclear Zr_6_O_8_ clusters and small cyclic ligands [16]. As shown in Scheme 1, the major difference is that in MOF-808, a Zr_6_O_8_ cluster is linked to 6 carboxylic acid ligands, whereas MOF-802 and UiO-66 Zr_6_O_8_ are linked to 10 ligands and 12 ligands, respectively. The crystalline structure and phase purity of the synthesized MOFs were thoroughly characterized using powder X-ray diffraction (PXRD), as depicted in Figure 1a. The observed characteristic diffraction peaks align well with the simulated patterns (Figures S1–S3), and the three different MOFs had great crystallinity. The N_2_ adsorption–desorption isotherms and pore size distribution curves of the three MOFs are shown in Figure 1b. Both MOF-808 and UiO-66 display a Type-I isotherm, which is characteristic of a microporous structure. MOF-802 exhibits type IV adsorption–desorption isotherms and displays a steep increase at a relatively low N_2_ partial pressure (P/P_0_ = 0–0.015), and the well-defined hysteresis loops at higher N_2_ pressures (P/P_0_ = 0.8–0.95), suggesting the coexistence of both micropores and mesopores in the samples. The specific Brunauer–Emmett–Teller surface areas and pore size distributions (Figures S5–S7) of MOF-808, MOF-802, and UiO-66 were similar to the previous report. Beyond that, the FT-IR spectra of MOF-808, MOF-802, and UiO-66 are shown in Figure S4 and the distinct absorption peaks corresponding to the ligands’ functional groups are clearly discernible. These results provide preliminary evidence for the successful acquisition of the three Zr-MOF materials.

**Figure 2:**
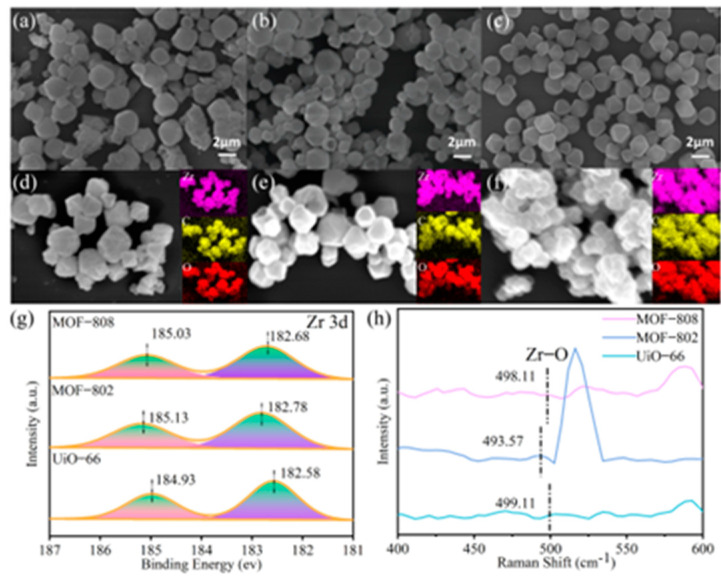
SEM images of the (**a**) MOF-808, (**b**) MOF-802, (**c**) UiO-66, and (**d**–**f**) the corresponding EDS mapping of Zr, C and O, (**g**) XPS characterization of Zr 3d MOF-808, MOF-802 and UiO-66 and (**h**) Raman spectra of MOF-808, MOF-802 and UiO-66.

The geometrical morphology of the obtained three Zr-MOFs were measured using a scanning electron microscope (SEM, JEOL JSM-7800F, Tokyo, Japan). As shown in Figure 2, MOF-808, MOF-802 and UiO-66 exhibited regular polyhedral morphology with a homogenous size of about 2 µm and can be visualized clearly (Figure S8). This also eliminates the interference of catalyst particle size in the catalysis experiment. In addition, the energy dispersive X-ray spectroscopy (EDS, LN2 Free SDD type and 150m^2^ type, Tokyo, Japan) elemental mapping characterization further confirmed the uniform distribution of Zr, C and O elements over all the three Zr-MOFs. To investigate elemental states of the Zr compound in the pore of three Zr-MOFs, X-ray photoelectron spectra (XPS) of three Zr-MOFs were recorded to determine its electronic states and elemental compositions. As depicted in Figure S9, XPS spectra of three Zr-MOFs suggest the existence of Zr, O and C. The high-resolution Zr 3d spectra of MOF-808 fitted into two peaks at 182.68 and 184.93 eV, which are attributed to Zr 3d_5/2_ and Zr 3d_3/2_ for Zr^4+^, respectively (Figure 2g). The peaks of Zr in MOF-802 are 182.78 eV and 185.13 eV, 182.58 eV and 185.03 eV in UiO-66, suggesting that the presence of Zr^4+^ in the Zr_6_O_8_ cluster [21]. The peaks results demonstrate that the Zr of the three catalysts is in the same chemical environment and the nuance is caused by differences in the electronegativity of the ligands and the number of ligands in the three materials, which is also illustrated by Raman spectroscopy in Figure 2h.

### 3.2. Investigation of the Effect of Different Linkage Numbers

**Figure 3:**
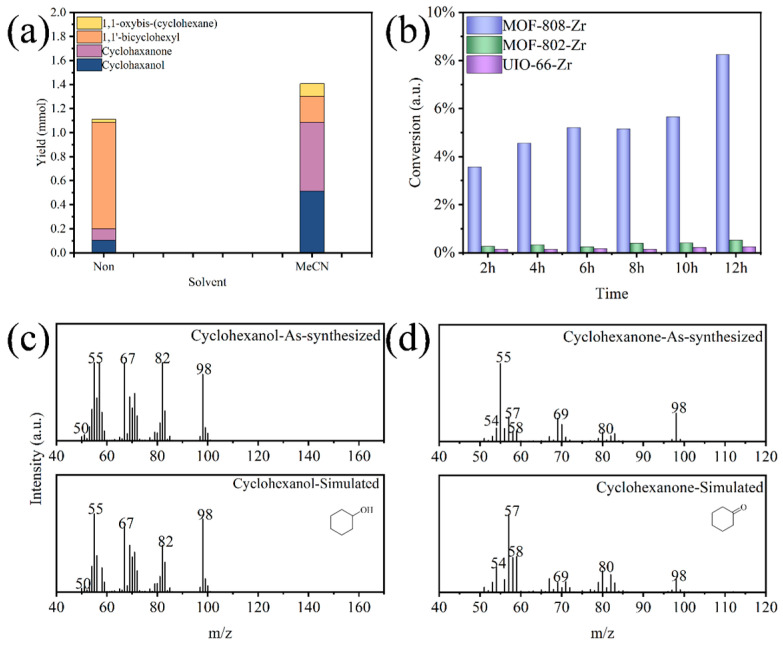
(**a**) Composition of products with and without solvents, (**b**) differences in catalytic performance of the three catalysts (reaction conditions: cyclohexane 1 mL, TBHP 1 mL, acetonitrile 4 mL, reaction temperature 70 °C), (**c**) GC-MS of experimental and standardized cyclohexanol and (**d**) GC-MS of experimental and standardized cyclohexanone.

To study the composition of post-catalysis reactions and facilitate quantitative analysis and provide evidence for mechanism inference, gas chromatography–mass spectrometry (GC-MS) component identification was first performed. The product yield of the solvent-free group was significantly different from that of the group with acetonitrile as the solvent, but the composition was consistent (Figure 3a). The GC-MS results of the reaction solution showed that the system contained cyclohexanol and cyclohexanone (collectively referred to as KA oil in industry), 1,1′-bicyclohexyl, 1,1-oxybis (cyclohexane), and the main byproduct of 1,2-epoxycyclooctane (Figures S10–S12). The signal of cyclohexyl hydrogen peroxide was also captured, which is the intermediate of cyclohexanol and cyclohexanone (Figure S13). Subsequently, the differences in the catalytic performance of MOFs with varying numbers of ligands were investigated (Figure 3b). As shown in Table 1, the 6-connected MOF-808 exhibited the most favorable catalytic performance achieving a high conversion of 8.30% and a TON value of 39.9 (Table 1, entry 1). The conversion and TON were significantly higher than that observed for the 10-connected MOF-802 of 0.53% and 3.27, respectively (Table 1, entry 2). Similarly, the conversion and TON were higher than that observed for the 12-connected UiO-66 of 0.36% and 2.24, respectively (Table 1, entry 3). In parallel, a comparison was conducted with previously reported catalysts, as illustrated in Table S1. The catalysts utilized in this study demonstrated effective catalytic performance under relatively mild conditions. Concurrently, the response selectivity was maintained at approximately 98% with consistent reliability.

**Figure 4:**
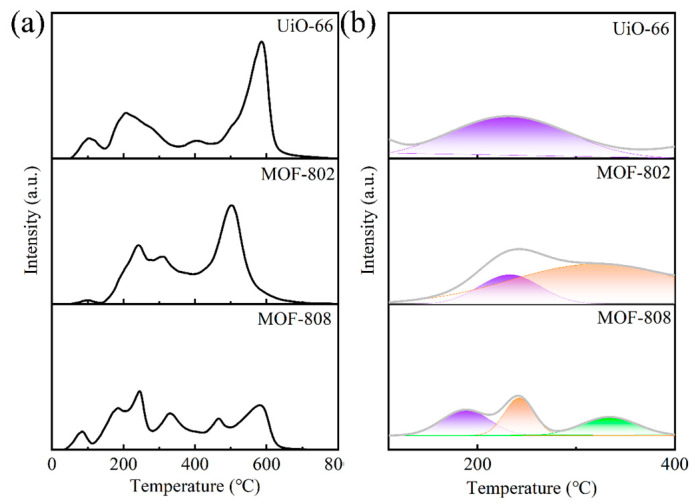
(**a**) TPD of cyclohexane with different catalysts and (**b**) cyclohexane desorption in the temperature range of 100–400 °C.

To ascertain the impact of the number of connections, a cyclohexane temperature-programmed desorption test (TPD) was conducted on catalysts. The combination of the thermogravimetric methods for the three MOFs revealed that the MOFs decompose at 400 °C, indicating that the desorption of cyclohexane occurs before this temperature (Figure S14). It was observed that the desorbed cyclohexane at 100 °C exhibited the characteristics of physically adsorbed cyclohexane. Therefore, we mainly consider parsing processes between 100 and 400 °C. As illustrated in Figure 4a, MOF-808 exhibits three distinct adsorption sites for cyclohexane, whereas UiO-66 displays a single adsorption site, and MOF-802 exhibits two adsorption sites for cyclohexane. In addition, to further understand the differences in the adsorption of cyclohexane by the catalyst, the curves in the range of 100–400 °C were integrated. It is evident that MOF-808 exerts the most pronounced chemical influence on cyclohexane (Figure 4b). This suggests that the number of linkages in Zr_6_O_8_ plays a significant role in influencing the adsorption of cyclohexane. It can be concluded that the amount of adsorption is a determining factor in the catalytic performance. The discrepancy in catalytic efficiency is mainly attributed to the quantity of active sites, which influences the accumulation of substrates.

**Figure 5:**
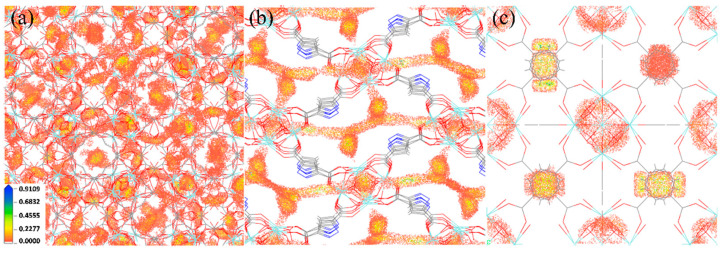
GCMC simulated density distributions of cyclohexane at 298 K and 1 bar for (**a**) MOF-808, (**b**) MOF-802 and (**c**) UiO-66.

A GCMC simulation was employed to calculate the potential adsorption sites and density distributions of guest cyclohexane molecules within the skeleton of three Zr-MOFs. As illustrated in Figure 5, the potential adsorption sites for cyclohexane in MOF-808 are primarily concentrated around and within the pores of 6-connected Zr_6_O_8_ clusters with open metal sites. In contrast, UiO-66 exhibits identical adsorption sites for cyclohexane as observed in MOF-802. The simulation results indicate that MOF-808 exhibits a stronger adsorption capacity from metal nodes in comparison to MOF-802 and UiO-66 [26,27]. This result is in accordance with the findings of the TPD test, which indicates that MOF-808 with 6-connected nodes exhibits the most effective catalytic effect, followed by MOF-802 and UiO-66. This calculation provides additional evidence for enhanced cyclohexane adsorption at unsaturated sites on the Zr_6_O_8_ framework, thereby improving overall catalytic performance.

### 3.3. Investigation of the Effect of Solvent and Temperature

**Figure 6:**
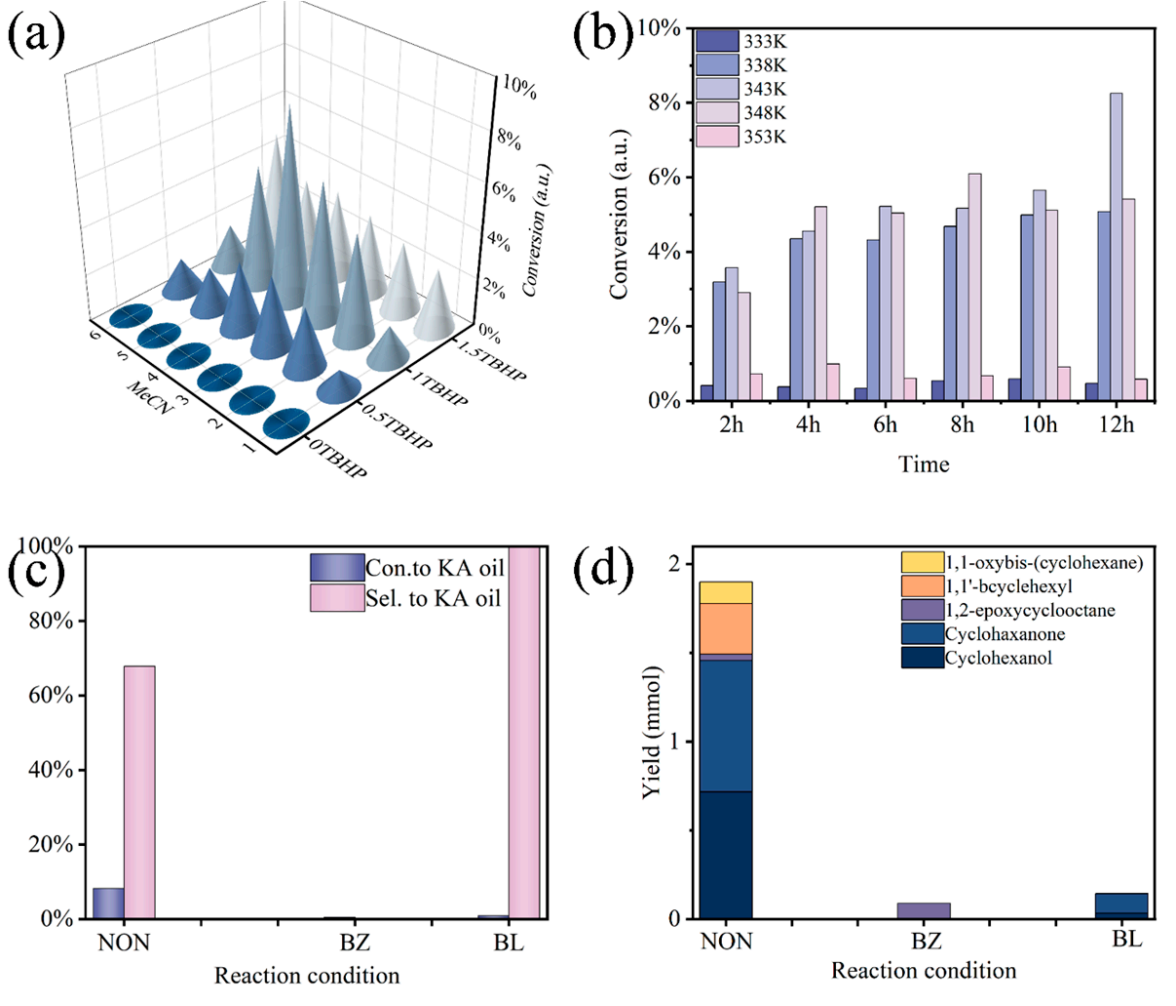
(**a**) Catalytic performance at 70 °C with different amounts of acetonitrile and TBHP, (**b**) catalysis of MOF-808 at different temperatures, (**c**) changes in conversion and selectivity before and after addition of the shielding agent and (**d**) composition of the product after the addition of the free-radical shielding agent (NON: without shielding agent, BZ: p-benzoquinone, BL: butanol).

MOF-808, which demonstrated the most effective catalytic effect, was selected for subsequent experiments to assess its potential for industrial application. It is noteworthy that previous reports have indicated that the quantity of acetonitrile employed is also a significant factor influencing the rate of conversion of cyclohexane [28,29]. In the absence of acetonitrile as a solvent, the production of cyclohexanol and cyclohexanone was diminished, and the product composition was primarily comprised of the R-R and R-O-R. Following the addition of acetonitrile, a change in product composition was observed, with an increase in the proportion of cyclohexanone and cyclohexanol. To ascertain the optimal ratio of acetonitrile to TBHP, the amount of acetonitrile and TBHP were varied in experimental studies (Figure 6a). As the quantity of acetonitrile increased, the conversion achieved its maximum when the value of acetonitrile was approximately four times that of TBHP. As the quantity of acetonitrile was further augmented, the selectivity of the system for KA oil increased (Figure S15), yet the conversion exhibited a decline. This is due to the fact that acetonitrile functions as a solvent within the system, and an adequate increase in dosage can effectively enhance the contact between cyclohexane and the catalyst. However, as the quantity of acetonitrile increased, the requisite contact between the catalyst and cyclohexane was insufficient, resulting in a decline in conversion. Moreover, the quantity of acetonitrile increased. The enhanced homogeneity of the R· and RO· dispersion may effectively diminish the contact between R· and RO·, consequently inhibiting the formation of 1,1′-bicyclohexane and 1,1-oxobis(cyclohexane) (Figure S16).

It is well established that temperature is a significant factor influencing the selectivity and conversion of reaction systems (Table 2). The conversion rates of MOF-808 at varying temperatures were investigated (Figure 6b). The conversion rate demonstrated an upward trajectory, followed by a decline, with an increase in temperature, reaching a peak at 70 °C. It can be attributed to a confluence of factors, including the boiling point of cyclohexane, the boiling point of acetonitrile, and the decomposition temperature of TBHP. As the temperature rises, the reaction accelerates. However, as the temperature approaches the boiling points of acetonitrile and cyclohexane, the conversion rate declines. Meanwhile, elevated temperatures impede the reaction of R· and RO·, thereby enhancing the selectivity of KA (Figure S17). This result provides compelling evidence that 1,1′-bicyclohexyne and 1,1-oxybis(cyclohexane) are generated by R· and RO·, and that the reaction follows a radical reaction course.

### 3.4. Free-Radical Capture Experiments

We then chose p-benzoquinone and butanol as free-radical shielding agents and found that the reaction conversion was reduced (Figure 6c). With the addition of p-benzoquinone, we could observe the epoxidation of cyclohexane, but we did not observe the generation of the by-product. On the other hand, when butanol was added to quench ·OH [30], it was observed that the products consisted of cyclohexanol and cyclohexanone, but no other products were found (Figure 6d). It is noteworthy that the conversion of cyclohexane decreased dramatically with the addition of a free-radical shielding agent. When shielding ${\cdot O}_{2}^{-}$, only a weak signal of the by-product 1,2-epoxyoctane was captured. Conversely, the conversion of cyclohexane decreases significantly when shielding ·OH. However, the product consists of only cyclohexanol and cyclohexanone. It suggests that ${\cdot O}_{2}^{-}$ plays a crucial role in initiating the reaction and shielding it leads to the low conversion of cyclohexane. In addition, ·OH is another important intermediate active species in the reaction and quenching it results in a small amount of cyclohexane conversion. Meanwhile, the catalyst always maintained a stable structure during this process (Figure S1).

### 3.5. Catalytic Reaction Mechanism

**Figure 7:**
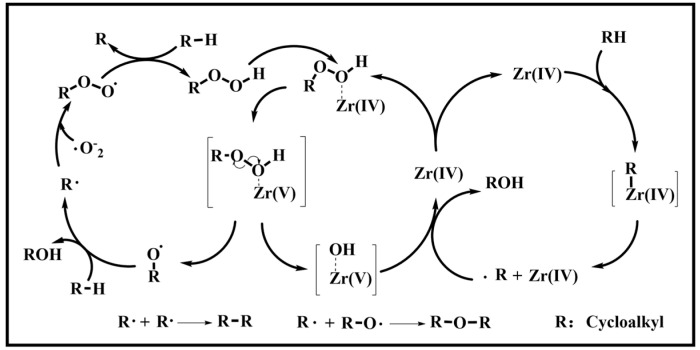
The mechanism of MOF-808 in the enhancement of cyclohexane oxidation.

In the reaction, an equal volume of cyclohexanol was introduced instead of cyclohexane. The formation of cyclohexanone was observed without the appearance of other by-products, indicating that cyclohexanone was oxidized from cyclohexanol and did not result in the formation of additional by-products (Figure S18). Thus, the mechanism of the cyclohexane oxidation using Zr-MOFs as catalysts was proposed (Figure 7) based on our results and previously reported literature [31,32,33]. Firstly, cyclohexane is adsorbed by the unsaturated node of Zr_6_O_8_, where it is activated to generate cyclohexyl radicals. These radicals are then oxidized by ${\cdot O}_{2}^{-}$ to generate ROO·, which further reacts with the cyclohexane molecule to generate ROOH and R·. This intermediate is then adsorbed by the unsaturated site of Zr_6_O_8_, where a bond breakage occurs to generate the hydroxyl radicals, which remain in Zr_6_O_8_. The generated RO· then reacts with RH to generate cyclohexanol and R·. On the other hand, the cyclohexyl radical is able to reduce Zr(V)–OH, resulting in the generation of a molecule of cyclohexanol and Zr (IV). This whole reaction completes a cycle, which explains the reason why the reactants were not converted after the ${\cdot O}_{2}^{-}$ was quenched. However, a minor quantity of cyclohexanol and cyclohexanone is still produced when selecting the scavenging of ·OH.

## 4. Conclusions

The difference effect of a series of catalysis cyclohexane oxidation reactions by three porous Zr(IV)-based MOFs of UiO-66, MOF-808, and MOF-802 was explored. It was found that a reduction in the Zr_6_O_8_ ligand linkage number resulted in an enhancement in the catalytic efficiency for cyclohexane oxidation. The main reason is that the MOFs with varying linkage numbers exhibited disparate enrichment capabilities for cyclohexane, which were confirmed by the TPD of cyclohexane, and also corroborated by GCMC simulation. Meanwhile, the MOF-802 catalysis effect was lower than expected, owing to its low porosity and narrow inner pore size. These results suggest that the discrepancy in the catalytic performance of Zr_6_O_8_ as an active site is primarily attributable to the variation in substrate enrichment capability. This study elucidates the internal factors that influence the relationship between the number of active sites and catalytic performance. Moreover, the catalyst’s potential for industrial application in the field of cyclohexane oxidation was demonstrated through the optimization of catalytic conditions.

## Figures and Tables

**Table 1:** Differences in catalytic performance of different catalysts (1 mL TBHP, 1 mL cyclohexane, 12 h, 70 °C and 1 bar O_2_).

Catalyst	Connected	Con.	Sel.	TON
MOF-808	6	8.30%	98%	39.9
MOF-802	10	0.53%	83%	3.27
UiO-66	12	0.36%	76%	2.24

**Table 2:** K-A oil formation after 12 h of reaction in the presence of MOF-808 (MOF-808: 0.015 mmol, 1 mL TBHP, 1 mL cyclohexane).

Temperature	Con.	Sel.		TON
		A	K	
60 °C	0.47%	38.93%	36.87%	2.93
65 °C	5.08%	41.65%	39.57%	31.59
70 °C	8.24%	31.06%	32.08%	39.28
75 °C	3.68%	29.13%	44.46%	22.74
80 °C	0.58%	20.54%	61.56%	3.53

## Data Availability

The original contributions presented in the study are included in the article/Supplementary Material and further inquiries can be directed to the corresponding authors.

## Supplementary Materials

The following supporting information can be downloaded at https://www.mdpi.com/article/10.3390/polym16223114/s1, Figure S1: The XRD of MOF-808: synthesized (black), after reaction (red) and simulated (blue); Figure S2: The XRD of MOF-802: synthesized (black) and simulated (red); Figure S3: The XRD of UiO-66: synthesized (black) and simulated (red); Figure S4: The IR spectra for MOF-808, MOF-802 and UiO-66; Figure S5: NLDFT-pore size distributions for MOF-808; Figure S6: NLDFT-pore size distributions for MOF-802; Figure S7: NLDFT-pore size distributions for UiO-66; Figure S8: Distribution of material sizes of MOF-808 (a), MOF-802 (b) and UiO-66 (c); Figure S9: The XPS full spectra for MOF-808 (black), MOF-802 (red) and UiO-66 (blue); Figure S10: GC-MS of 1,1′-bicyclohexyl; Figure S11: GC-MS of 1,1-oxybis (cyclohexane); Figure S12: GC-MS of 1,2-epoxycyclooctane; Figure S13: GC-MS of cyclohexyl hydrogen peroxide; Figure S14: Thermogravimetric analysis for MOf-808, MOF-802 and UiO-66; Figure S15: Cyclohexane 1 mL, temperature 70 °C, 1 bar O_2_ and variation in cyclohexane-catalyzed reaction selectivity with the amount of acetonitrile; Figure S16: Cyclohexane 1 mL, acetonitrile 4 mL, 1 bar O_2_, cyclohexane-catalyzed reaction selectivity versus time at different temperatures; Figure S17: Product composition of pure cyclohexane with acetonitrile as solvent at 70 °C, 1 bar O_2_; Figure S18: Cyclohexanol as a substrate product over time at 70 °C, 1 bar O_2_; Table S1: Comparison of reported performance for cyclohexane-catalyzed (Cyc: indicates the amount of cyclohexane). References [34,35,36,37,38] are included in the Supplementary Materials.

## References

[1] Zhang X., Chen Z., Liu X., Hanna S.L., Wang X., Taheri-Ledari R., Maleki A., Li P., Farha O.K. (2020). A historical overview of the activation and porosity of metal-organic frameworks. Chem. Soc. Rev..

[2] Xu C., Pan Y., Wan G., Liu H., Wang L., Zhou H., Yu S.H., Jiang H.L. (2019). Turning on Visible-Light Photocatalytic C-H Oxidation over Metal-Organic Frameworks by Introducing Metal-to-Cluster Charge Transfer. J. Am. Chem. Soc..

[3] Sun Z.B., Si Y.N., Zhao S.N., Wang Q.Y., Zang S.Q. (2021). Ozone Decomposition by a Manganese-Organic Framework over the Entire Humidity Range. J. Am. Chem. Soc..

[4] Dong L.Z., Zhang L., Liu J., Huang Q., Lu M., Ji W.X., Lan Y.Q. (2020). Stable Heterometallic Cluster-Based Organic Framework Catalysts for Artificial Photosynthesis. Angew. Chem. Int. Ed..

[5] Gao P., Mukherjee S., Zahid Hussain M., Ye S., Wang X., Li W., Cao R., Elsner M., Fischer R.A. (2024). Porphyrin-based MOFs for sensing environmental pollutants. Chem. Eng. J..

[6] Lin Y., Li W.H., Wen Y., Wang G.E., Ye X.L., Xu G. (2021). Layer-by-Layer Growth of Preferred-Oriented MOF Thin Film on Nanowire Array for High-Performance Chemiresistive Sensing. Angew. Chem. Int. Ed..

[7] Kasper T., Pavan M., Müller-Buschbaum K. (2024). On the validity of rapid optical sensing of dioxygen by means of sensitivity, stability, and reversibility for archetype MOFs post-synthetically modified with Eu^3+^. J. Mater. Chem. A.

[8] Li H., Wang K., Sun Y., Lollar C.T., Li J., Zhou H.C. (2018). Recent advances in gas storage and separation using metal-organic frameworks. Mater. Today.

[9] Han Y., Liu L., Han Z., Wu M. (2024). Engineering ethane-trapping metal-organic framework for efficient ethylene separation under high humid conditions. Sep. Purif. Technol..

[10] Lin S., Zhao Y., Yun Y.S. (2018). Highly Effective Removal of Nonsteroidal Anti-Inflammatory Pharmaceuticals from Water by Zr(IV)-Based Metal-Organic Framework: Adsorption Performance and Mechanisms. ACS Appl. Mater. Interfaces.

[11] Mahmoodi N.M., Abdi J., Oveisi M., Alinia Asli M., Vossoughi M. (2018). Metal-organic framework (MIL-100 (Fe)): Synthesis, detailed photocatalytic dye degradation ability in colored textile wastewater and recycling. Mater. Res. Bull..

[12] Zhao F., Liu Y., Hammouda S.B., Doshi B., Guijarro N., Min X., Tang C.J., Sillanpää M., Sivula K., Wang S. (2020). MIL-101(Fe)/g-C3N4 for enhanced visible-light-driven photocatalysis toward simultaneous reduction of Cr(VI) and oxidation of bisphenol A in aqueous media. Appl. Catal. B Environ..

[13] Jiang H., Wang Q., Wang H., Chen Y., Zhang M. (2016). MOF-74 as an Efficient Catalyst for the Low-Temperature Selective Catalytic Reduction of NOx with NH_3_. ACS Appl. Mater. Interfaces.

[14] Andrade P.H.M., Henry N., Volkringer C., Loiseau T., Vezin H., Hureau M., Moissette A. (2022). Iodine Uptake by Zr-/Hf-Based UiO-66 Materials: The Influence of Metal Substitution on Iodine Evolution. ACS Appl. Mater. Interfaces.

[15] Bon V., Senkovska I., Baburin I.A., Kaskel S. (2013). Zr- and Hf-Based Metal-Organic Frameworks: Tracking Down the Polymorphism. Cryst. Growth Des..

[16] Furukawa H., Gándara F., Zhang Y.B., Jiang J., Queen W.L., Hudson M.R., Yaghi O.M. (2014). Water Adsorption in Porous Metal-Organic Frameworks and Related Materials. J. Am. Chem. Soc..

[17] Lu Z., Liu J., Zhang X., Liao Y., Wang R., Zhang K., Lyu J., Farha O.K., Hupp J.T. (2020). Node-Accessible Zirconium MOFs. J. Am. Chem. Soc..

[18] Morris W., Volosskiy B., Demir S., Gándara F., McGrier P.L., Furukawa H., Cascio D., Stoddart J.F., Yaghi O.M. (2012). Synthesis, Structure, and Metalation of Two New Highly Porous Zirconium Metal-Organic Frameworks. Inorg. Chem..

[19] Wang J., Fan Y., Tan Y., Zhao X., Zhang Y., Cheng C., Yang M. (2018). Porphyrinic Metal-Organic Framework PCN-224 Nanoparticles for Near-Infrared-Induced Attenuation of Aggregation and Neurotoxicity of Alzheimer’s Amyloid-β Peptide. ACS Appl. Mater. Interfaces.

[20] Ji P., Drake T., Murakami A., Oliveres P., Skone J.H., Lin W. (2018). Tuning Lewis Acidity of Metal-Organic Frameworks via Perfluorination of Bridging Ligands: Spectroscopic, Theoretical, and Catalytic Studies. J. Am. Chem. Soc..

[21] Fang G., Hu J.N., Tian L.C., Liang J.X., Lin J., Li L., Zhu C., Wang X. (2022). Zirconium-oxo Nodes of MOFs with Tunable Electronic Properties Provide Effective OH Species for Enhanced Methane Hydroxylation. Angew. Chem. Int. Ed..

[22] Vomeri A., Stucchi M., Villa A., Evangelisti C., Beck A., Prati L. (2022). New insights for the catalytic oxidation of cyclohexane to K-A oil. J. Energy Chem..

[23] Tao Y., Yang B., Wang F., Yan Y., Hong X., Xu H., Xia M., Wang F. (2022). Green synthesis of MOF-808 with modulation of particle sizes and defects for efficient phosphate sequestration. Sep. Purif. Technol..

[24] Zhang J., He X., Kong Y.R., Luo H.B., Liu M., Liu Y., Ren X.M. (2021). Efficiently Boosting Moisture Retention Capacity of Porous Superprotonic Conducting MOF-802 at Ambient Humidity via Forming a Hydrogel Composite Strategy. ACS Appl. Mater. Interfaces.

[25] Lin S., Zhao Y., Bediako J.K., Cho C.W., Sarkar A.K., Lim C.R., Yun Y.S. (2019). Structure-controlled recovery of palladium(II) from acidic aqueous solution using metal-organic frameworks of MOF-802, UiO-66 and MOF-808. Chem. Eng. J..

[26] Lv H.J., Zhang J.W., Jiang Y.C., Li S.N., Hu M.C., Zhai Q.G. (2022). Micropore Regulation in Ultrastable [Sc3O]-Organic Frameworks for Acetylene Storage and Purification. Inorg. Chem..

[27] Yao J., Zhao Z., Yu L., Huang J., Shen S., Zhao S., Wu Y., Tian X., Wang J., Xia Q. (2023). Boosting trace SO_2_ adsorption and separation performance by the modulation of the SBU metal component of iron-based bimetal MOFs. J. Mater. Chem. A.

[28] He X., Looker B.G., Dinh K.T., Stubbs A.W., Chen T., Meyer R.J., Serna P., Román-Leshkov Y., Lancaster K.M., Dincă M. (2020). Cerium(IV) Enhances the Catalytic Oxidation Activity of Single-Site Cu Active Sites in MOFs. ACS Catal..

[29] Li L., Yang Q., Chen S., Hou X., Liu B., Lu J., Jiang H.L. (2017). Boosting selective oxidation of cyclohexane over a metal-organic framework by hydrophobicity engineering of pore walls. Chem. Commun..

[30] Guo Y., Long J., Huang J., Yu G., Wang Y. (2022). Can the commonly used quenching method really evaluate the role of reactive oxygen species in pollutant abatement during catalytic ozonation?. Water Res..

[31] Shen H.M., Wang X., Ning L., Guo A.B., Deng J.H., She Y.B. (2021). Efficient oxidation of cycloalkanes with simultaneously increased conversion and selectivity using O_2_ catalyzed by metalloporphyrins and boosted by Zn(AcO)_2_: A practical strategy to inhibit the formation of aliphatic diacids. Appl. Catal. A Gen..

[32] He H.H., Yuan J.P., Cai P.Y., Wang K.Y., Feng L., Kirchon A., Li J., Zhang L.L., Zhou H.-C., Fang Y. (2023). Yolk-Shell and Hollow Zr/Ce-UiO-66 for Manipulating Selectivity in Tandem Reactions and Photoreactions. J. Am. Chem. Soc..

[33] Montjoy D.G., Wilson E.A.K., Hou H., Graves J.D., Kotov N.A. (2023). Photocatalytic cyclohexane oxidation and epoxidation using hedgehog particles. Nat. Commun..

[34] Wang S., Sun Z., Zou X., Zhang Z., Fu G., Li L., Zhang X., Luo F. (2019). Enhancing catalytic aerobic oxidation performance of cyclohexaneviasize regulation of mixed-valence {V16} cluster-based metal–organic frameworks. New J. Chem..

[35] Wang Y., Zhao L., Ji G., He C., Liu S., Duan C. (2022). Vanadium(V^IV^)–Porphyrin-Based Metal–Organic Frameworks for Synergistic Bimetallic Activation of Inert C(sp^3^)–H Bonds. ACS Appl. Mater. Interfaces.

[36] Liu Y., Tsunoyama H., Akita T., Xie S., Tsukuda T. (2010). Aerobic Oxidation of Cyclohexane Catalyzed by Size-Controlled Au Clusters on Hydroxyapatite: Size Effect in the Sub-2 nm Regime. ACS Catal..

[37] Li X.-H., Chen J.-S., Wang X., Sun J., Antonietti M. (2011). Metal-Free Activation of Dioxygen by Graphene/g-C_3_N_4_Nanocomposites: Functional Dyads for Selective Oxidation of Saturated Hydrocarbons. J. Am. Chem. Soc..

[38] Ji G., Zhao L., Wang Y., Tang Y., He C., Liu S., Duan C. (2022). A Binuclear Cerium-Based Metal–Organic Framework as an Artificial Monooxygenase for the Saturated Hydrocarbon Aerobic Oxidation with High Efficiency and High Selectivity. ACS Catal..

