# Supplementary material for: Investigation into the Internal Factors for the Catalytic Oxidation of Cyclohexane by Zr(IV)-Based Metal-Organic Frameworks
Source: Polymers (Basel). 2024 Nov 6;16(22):3114. doi: 10.3390/polym16223114 (PMC11598140; doi:10.3390/polym16223114)
Supplement: Supplementary file 1 [file polymers-16-03114-s001.zip › polymers-3260067-supplementary.pdf]

Electronic Supplementary Information

XPS Setting parameters:

An X-ray photoelectron spectrometer (ThermoFischer, ESCALAB 250Xi, USA) was used for this experiment. In particular, the vacuum of the analyzing chamber was 4xl0^{-9} mbar, the excitation source was Al ka ray (hv=1486.6 eV), the operating voltage was 14.6 kV, the filament current was 13.5 mA, and the signal was accumulated for 20 cycles. The test pass energy (Passing-Energy) was 20 ev with a step of 0.1 eV, and the charge correction was performed with C1s=284.8 eV binding energy as the energy standard.

Etching conditions (no etching, negligible): the sample was etched and thinned using an argon ion gun with an etching spot size of 1.5 mm, an etching voltage of 3000 eV, and an etching rate of 0.05 nm/s. Regular etching was performed for about 100 s.

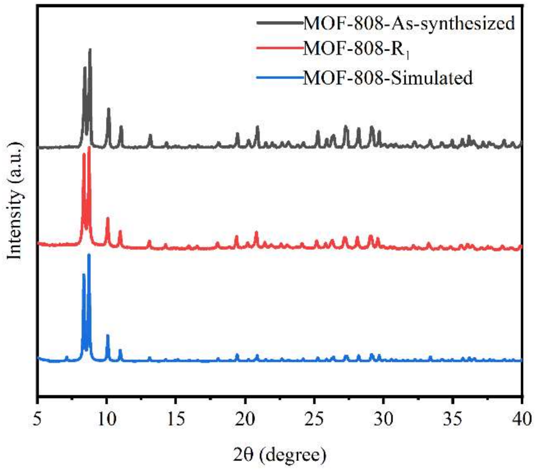

Fig. S1. The XRD of MOF-808, synthesized(black), after reaction(red), simulated(blue).

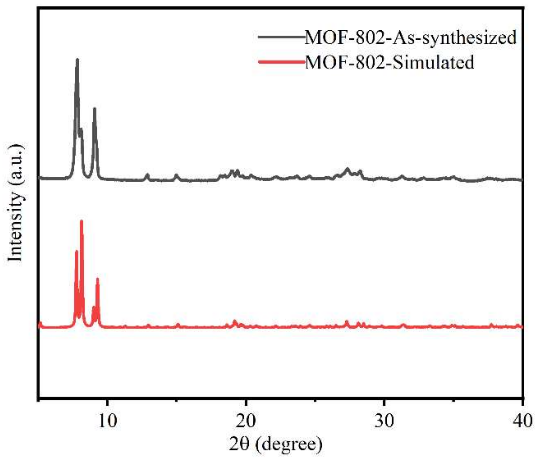

Fig. S2. The XRD of MOF-802, synthesized(black), simulated(red).

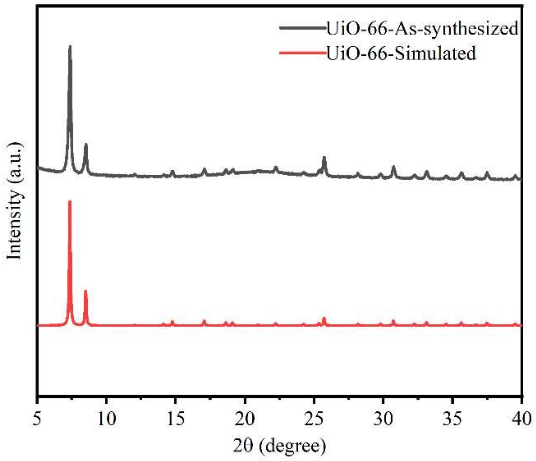

Fig. S3. The XRD of UiO-66, synthesized(black), simulated(red).

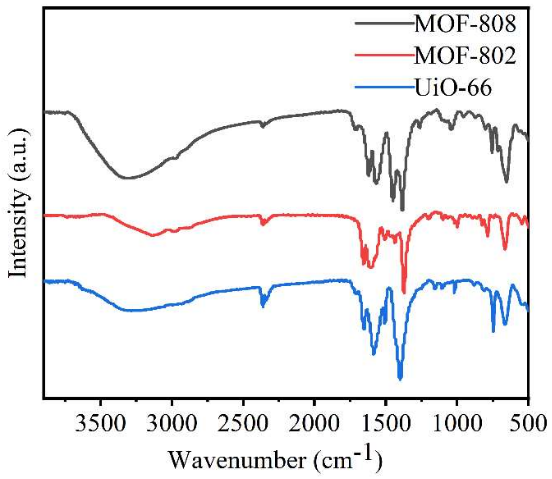

Fig. S4. The IR spectra for MOF-808, MOF-802 and UiO-66.

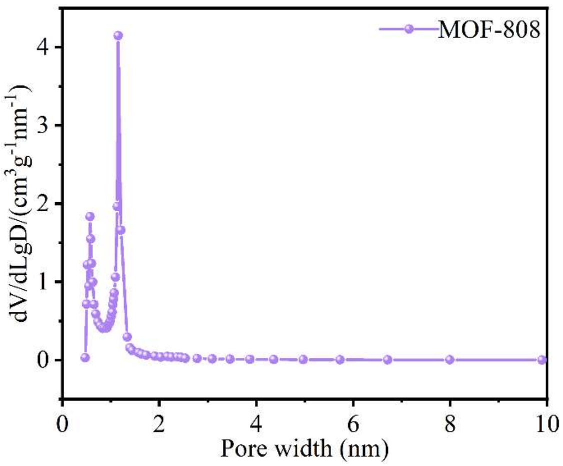

Fig. S5. NLDFT-pore size distributions for MOF-808.

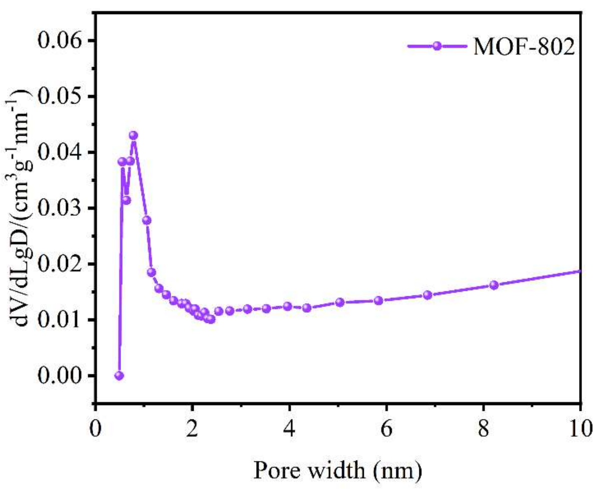

Fig. S6. NLDFT-pore size distributions for MOF-802.

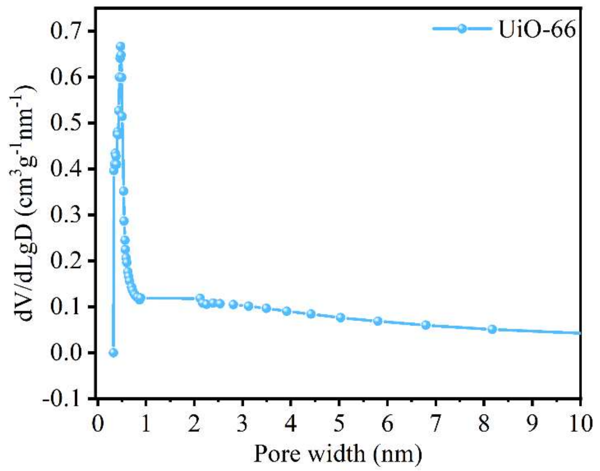

Fig. S7. NLDFT-pore size distributions for UiO-66.

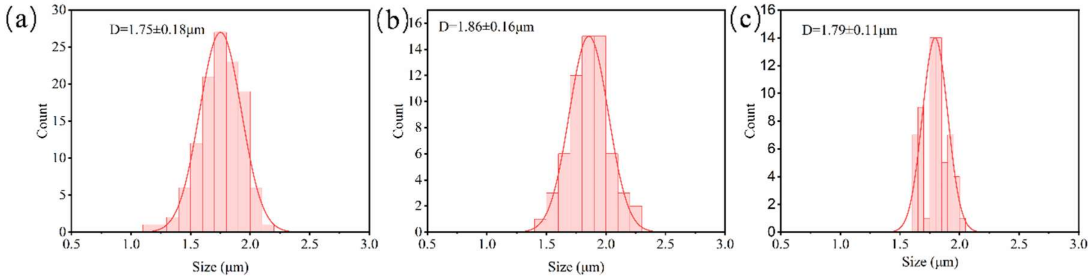

Fig. S8. Distribution of material sizes of MOF-808(a), MOF-802(b) and UiO-66(c).

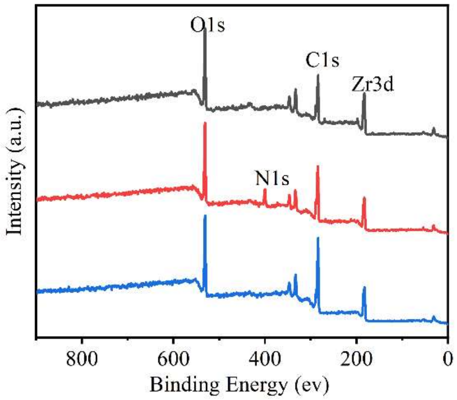

Fig. S9. The XPS full spectra for MOF-808(black), MOF-802(red)and UiO-66(blue).

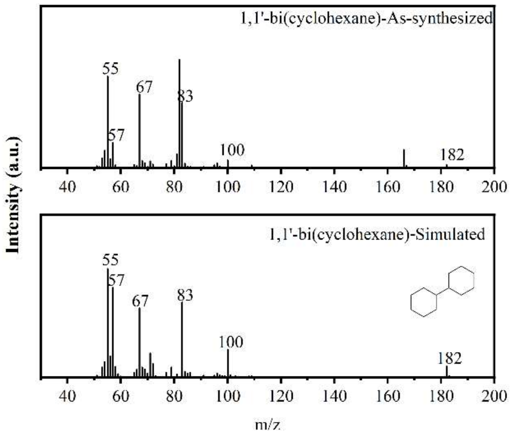

Fig. S10. GC-MS of 1,1'-bicyclohexyl.

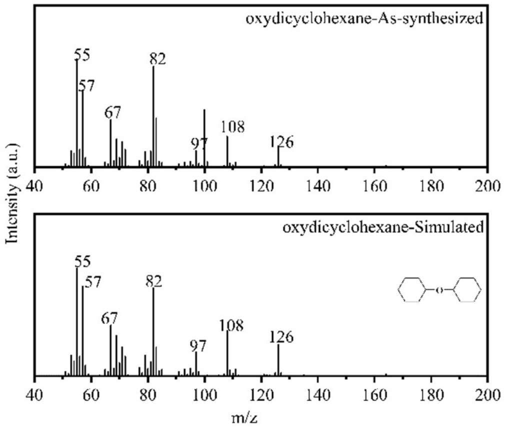

Fig. S11. GC-MS of 1,1-oxybis (cyclohexane).

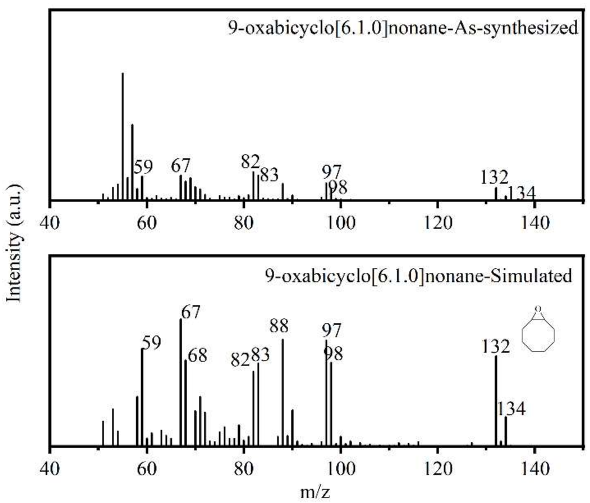

Fig. S12.GC-MS of 1,2-epoxycyclooctane.

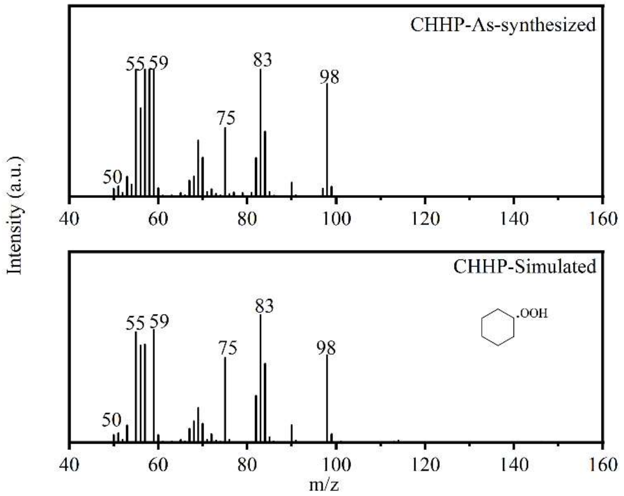

Fig. S13.GC-MS of cyclohexyl hydrogen peroxide.

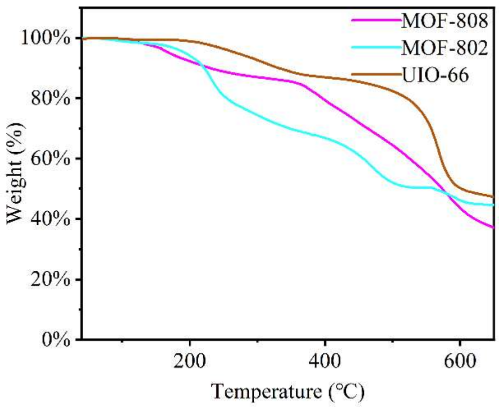

Fig. S14 Thermogravimetric analysis for MOf-808, MOF-802 and UiO-66.

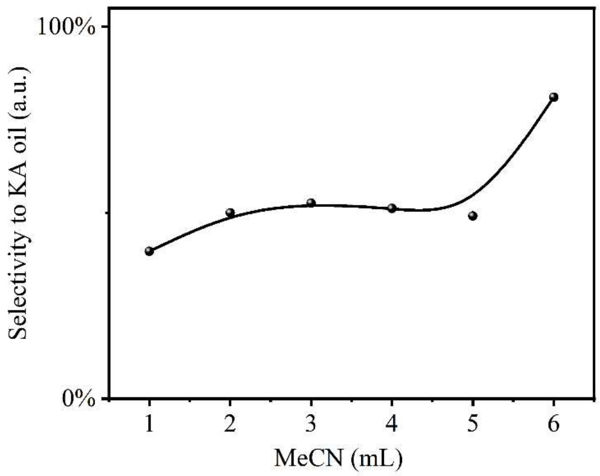

Fig. S15. Cyclohexane 1 mL, temperatures 70 ºC, 1 bar O_2, variation of cyclohexane-catalysed reaction selectivity with the amount of acetonitrile.

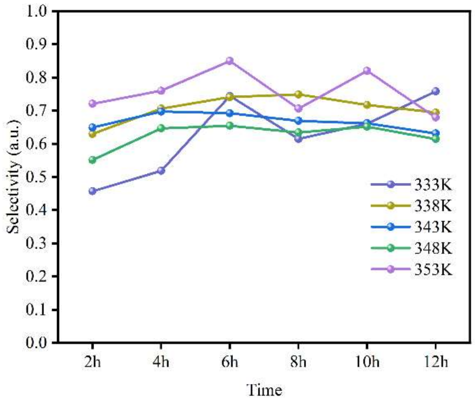

Fig. S16. Cyclohexane 1 mL, acetonitrile 4 mL, 1 bar O_2, cyclohexane-catalyzed reaction selectivity versus time at different temperatures.

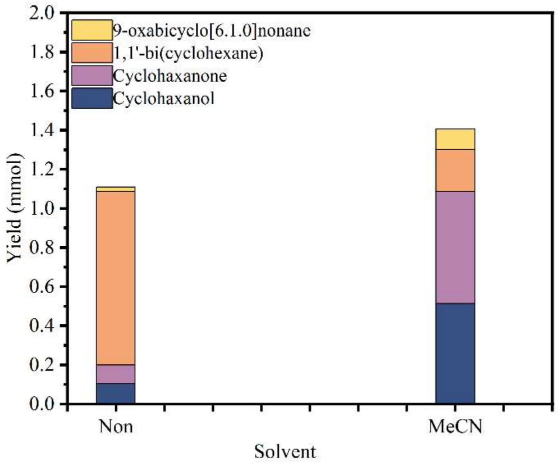

Fig. S17. Product composition of pure cyclohexane with acetonitrile as solvent at 70 °C, 1 barO_2.

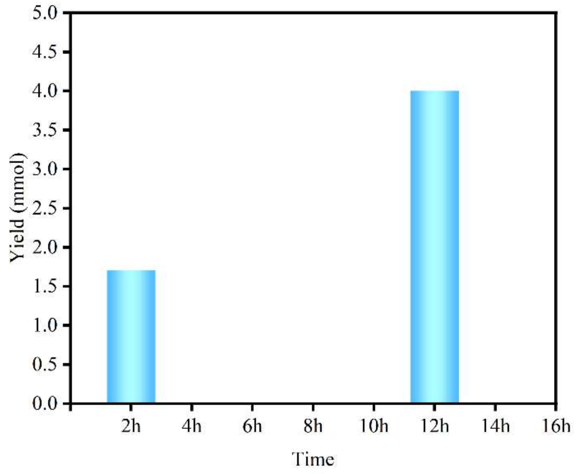

Fig. S18. Cyclohexanol as a substrate product over time at 70 ºC, 1 barO_2.

Table S1 Comparison of reported performance for cyclohexane-catalyzed (Cyc: indicates the amount of cyclohexane).

Catalyst	Cyc.	Condition	Con.	Sel.	Ref.
NENU-MV	5 mL	150 ^{O}C<br>1 Mbar O_2	24.6%	99%	[1]
Mn-TCPP(V)	0.5 mL	lamp	0.20%	58%	[2]
Aun/HA	10mL	150 ^{O}C<br>1 Mbar O_2	14%	99%	[3]
Co/g-C_3N_4	30 mL	130 ^{O}C<br>0.8 Mbar<br>O_2	12%	99%	[4]
Ce-AQ	0.01 mL	lamp	53.76%	98.40%	[5]
MOF-808	0.5 mL	70 ^{O}C<br>1 bar O_2	8.25%	98%	This work

[1] S. Wang, Z. Sun, X. Zou, Z. Zhang, G. Fu, L. Li, X. Zhang, F. Luo, Enhancing catalytic aerobic oxidation performance of cyclohexaneviasize regulation of mixed-valence {V16} cluster-based metal–organic frameworks, New Journal of Chemistry 43(36) (2019) 14527-14535.

[2] Y. Wang, L. Zhao, G. Ji, C. He, S. Liu, C. Duan, Vanadium(VIV)–Porphyrin-Based Metal–Organic Frameworks for Synergistic Bimetallic Activation of Inert C(sp3)–H Bonds, ACS Applied Materials & Interfaces 14(2) (2022) 2794-2804.

[3] Y. Liu, H. Tsunoyama, T. Akita, S. Xie, T. Tsukuda, Aerobic Oxidation of Cyclohexane Catalyzed by Size-Controlled Au Clusters on Hydroxyapatite: Size Effect in the Sub-2 nm Regime, ACS Catalysis 1(1) (2010) 2-6.

[4] X.-H. Li, J.-S. Chen, X. Wang, J. Sun, M. Antonietti, Metal-Free Activation of Dioxygen by Graphene/g-C3N4Nanocomposites: Functional Dyads for Selective Oxidation of Saturated Hydrocarbons, Journal of the American Chemical Society 133(21) (2011) 8074-8077.

[5] G. Ji, L. Zhao, Y. Wang, Y. Tang, C. He, S. Liu, C. Duan, A Binuclear Cerium-Based Metal–Organic Framework as an Artificial Monooxygenase for the Saturated Hydrocarbon Aerobic Oxidation with High Efficiency and High Selectivity, ACS Catalysis 12(13) (2022) 7821-7832.
